# Anesthesia characteristic of an algorithm of bupivacaine dose based on height in caesarean section under spinal anesthesia: a retrospective cohort study

**DOI:** 10.1186/s12871-023-02113-0

**Published:** 2023-05-02

**Authors:** Jinxin Huang, Gengzhi Wen, Qiang Huang, Bowan Huang

**Affiliations:** 1 Shenzhen Traditional Chinese Medicine Hospital, The Fourth Clinical Medical College of Guangzhou University of Chinese Medicine, Shenzhen City, Guangdong 518033 PR China; 2 Department of Anesthesiology, Shenzhen Traditional Chinese Medicine Hospital, The Fourth Clinical Medical College of Guangzhou University of Chinese Medicine, Shenzhen City, Guangdong 518033 PR China; 3 Department of Anesthesiology, ShenZhen People’s Hospital, 2nd Clinical Medical College of Jinan University, Shenzhen City, Guangdong 518020 PR China

**Keywords:** Anesthesia characteristic, Anesthesia, spinal, Bupivacaine, Cesarean section, Height

## Abstract

**Background:**

An algorithm of bupivacaine dose based on height is applied to reduce maternal hypotension in caesarean section under spinal anesthesia. This study is designed to further verify whether the algorithm of bupivacaine dose based on height is suitable.

**Methods:**

The parturients were grouped according to height. The comparison of anesthesia characteristic among subgroups was carried out. The univariate and multivariate binary logistic regressions were executed to reanalyze the interference factor for the anesthesia characteristic.

**Results:**

When the dose of bupivacaine was adjusted by using the height based dosing algorithm, except for weight (*P* < 0.05), other general data did not present statistical changes with height (*P* > 0.05); the incidences of complications, characteristics of sensory or motor block, quality of anesthesia and neonatal outcome were of no statistical difference among parturients with different heights (*P* > 0.05); the height, weight and body mass index were not related with maternal hypotension (*P* > 0.05). When the dose of bupivacaine is constant, except for weight and body mass index (*P* > 0.05), the height was the independent risk factor for maternal hypotension (*P* < 0.05).

**Conclusions:**

Except for weight and body mass index, the height has an influence on the bupivacaine dose. It is reasonable that the bupivacaine dose is adjusted by using this dosing algorithm based on height.

**Trial Registration:**

This study was registered at http://clinicaltrials.gov (13/04/2018, NCT03497364).

## Background

In previous study, we preliminarily verify that the bupivacaine dose depends on the parturient’s height in caesarean section under spinal anesthesia [1], but which needs to be further investigated. In addition, we have develop an algorithm of bupivacaine dose based on height in spinal anesthesia, which can provide sufficient anesthesia, but induce a low occurrence rate of hypotension in the parturients without prophylactic vasopressors and fluid preloading [1]. However, the anesthesia characteristic of this algorithm is not thoroughly clear, and it is unclear whether this algorithm is the optimal algorithm. Solving the above problems helps to improve the quality of spinal anesthesia in caesarean section.

In spinal anesthesia, the association between parturient’s height and local anesthetic’s dose is controversial. A study presents that the height is not statistically related with the block level [2]. In many studies, the local anesthetic’s dose is constant in parturients with different heights [3–6]. However, the height is associated with vertebral column length [7], which has an influence in the block level [8]. Thus, the local anesthetic’s dose should have an association with the height, which is confirmed by two studies [1, 9]. In our last article, when using a constant bupivacaine dose, the shorter parturients have a higher occurrence rate of hypotension, a faster sensory block time or a higher proportion of complete motor block [1]. When the bupivacaine dose is altered based on height, the occurrence rate of hypotension, sensory block time or proportion of complete motor block is similar in parturients with different heights [1]. However, the baseline data is not analyzed [1]. It is possible that there are other variables, which may interfere with the comparison of hypotension or sensory and motor blocks in parturients with different heights. In addition, the more detailed data (e.g., other complications, neonatal outcome and quality of anesthesia) of different height parturients are not compared [1].

In this study, we retrospectively analyzed the data of parturients, which underwent caesarean section under spinal anesthesia (the bupivacaine dose is constant or is based on height) [1]. In this manner, we attempted to further ascertain the association between parturient’s height and local anesthetic’s dose, and anesthesia characteristic of the algorithm of bupivacaine dose based on height in caesarean section under spinal anesthesia.

## Methods

### General

The Ethics Committee of Shenzhen People’s Hospital of Jinan University approved this study protocol. On 13/04/2018, we enrolled this study at ClinicalTrials.gov (NCT03497364). The study protocol was published in BMJ Open [10]. This study was performed in compliance with ICH-GCP and the Helsinki Declaration at the Department of Anesthesiology of Shenzhen People’s Hospital of Jinan University. Two hundred and fifty-eight parturients (18–45 years old, prepared for cesarean section) were included, and were randomly allocated to the Test or Conventional group by a research assistant (blocked randomization, 1:1 ratio). The parturients, outcomes assessors and surgical team were blinded to the randomization information. For all parturients, non-fatty solids intake was forbade for 6–8 h, and clear liquids intake was forbade for 2–3 h. From all parturients, written informed consent was obtained. The parturients who had contraindications of combined spinal-epidural anesthesia (CSE), fetal abnormalities, multiple births, placental abnormalities, systolic blood pressure (SBP) which was lower than 90 mmHg, cardiovascular disease and pre-eclampsia, were not included in this study [1].

### Intervention

In obstetric ward, the parturients’ blood pressure and heart rate (HR) were first obtained as the baseline values. Then, in the operation room, the HR, blood pressure, SPO_2_ and electrocardiogram were measured. Via a facemask, oxygen (2 L/min) was offered. In the vein in forearm, venous catheterization was performed. Next, Ringer’s lactate was given to the parturients in both groups at an infusion rate of 2 ml/kg/h.

In this study, spinal anesthesia is replaced with CSE. In left lateral position, at the L3-4 interspace, CSE was performed. In the Test group, 0.5% bupivacaine (1.15–1.7 ml, isobaric) was injected (0.05 ml/2–3 cm) [10], which was bought from ChaoHui drug company of ShangHai in China. The bupivacaine dose was based on the parturients’ height. In the Conventional group, 0.5% bupivacaine (1.8 ml, isobaric) was injected. On spinal needle in both groups, the side opening’s direction was towards the head. After injecting bupivacaine, the parturients were immediately adjusted to a 15° left lateral tilt position. The infusion rate of Ringer’s lactate was adjusted to 10 ml/kg/h in both groups [11]. In the Conventional group, phenylephrine (2.5 ml/h (i.e., 0.25 µg.kg^−1^.min^−1^)) was prophylactically given via micropump [12]. In the Test group, normal saline (2.5 ml/h) was given. For all participants, prophylactic fluid preloading was not performed.

SBP < 90 mmHg or 70% of baseline value was the criteria for judging maternal hypotension. If maternal hypotension happened from anesthesia initiation to delivery, this parturient was considered as a hypotensive parturient. Phenylephrine (100 µg) was used to treat maternal hypotension. Atropine (0.5 mg) was used to treat HR < 60 beat/min. Metoclopramide (10 mg) was used to treat nausea and vomiting. Based on anatomical structure, it is required that the highest level of sensory block is not lower than dermatome level dominated by the fourth thoracic nerve (T4) for caesarean Sect. [13]. However, the T4, T5, T6 or T8 sensory block level is required for adequate anesthesia in different studies [5, 14–18]. Even if the highest level of sensory block is higher than T4, some parturients still complain slight pain [13]. In our last article, the level of sensory block is required to reach T8 at 10 min after anesthesia [1]. Actually, the level of sensory block can reach T6 in most parturients at 10 min after anesthesia; the time from anesthesia initiation to skin incision is 19.024 (7.411) min; the quality of anesthesia in Test group is not statistically different from it in Conventional group [1]. Therefore, it is reasonable that when the level of sensory block was lower than T8 at 10 min after anesthesia, it was deemed to a failure [16, 17]. The parturients with unsuccessful spinal anesthesia were not included in this study. In parturients with unsuccessful spinal anesthesia, a 15 ml of 2% lidocaine + 0.75% ropivacaine was injected via epidural catheter until the level of sensory block was higher than or equal to T8, or the CSE was replaced with general anesthesia. If the parturients with successful spinal anesthesia complained pain after delivery, fentanyl (0.1 mg, vein) and/or 2% lidocaine + 0.75% ropivacaine (15 ml, epidural space) were performed.

### Data acquisition

Before spinal anesthesia, the general data was noted. After injecting bupivacaine, the HR, blood pressure, SPO_2_ and respiratory rate were intermittently measured. The sensory block level (hypoalgesia) was evaluated. If at 10 min after anesthesia, the hypoalgesia level was higher than or equal to T8, the anesthesia was deemed to be adequate [16, 17]. The motor block was measured via the modified Bromage scale [18].

At 1 and 5 min after the fetus was took out, APGAR scores were evaluated. The blood sample of umbilical artery was collected for blood gas analysis. The complications (i.e., hypotension (primary outcome), bradycardia, nausea, vomiting, dyspnea and dizziness) were noted.

After operation, the time from anesthesia initiation to skin incision, time from skin incision to delivery and operation duration were calculated. The quality of analgesia, the quality of muscle relaxation and the degree of intraoperative comfort were respectively evaluated by the anesthetist, surgeon and parturient, and were labeled as excellent, good, fair or poor [5].

### Statistical analysis

The methods for sample size calculation could be obtained in our last article [1]. Data analysis was carried out via SPSS 13.0 software. The continuous data were showed with the mean (SD). The enumeration data were compared with Chi-square test. The continuous data were compared with one-way ANOVA (Normally distributed data) or K Independent Samples Test (Non-normally distributed data, Kruskal–Wallis H-test). The univariate and multivariate binary logistic regressions were applied to analyze the influences of height, weight and body mass index (BMI) in maternal hypotension. *P* < 0.05 or 0.10 was considered significant.

## Results

After 13/04/2018, the parturients were recruited until the sample size was sufficient. The Test group had 127 parturients, and the Conventional group had 131 parturients [1]. These parturients in Test group were divided into six subgroups according to the height (Group_(145–149)_, Group_(150–154)_, Group_(155–159)_, Group_(160–164)_, Group_(165–169)_ and Group_(170–174)_). The Group_(145–149)_, Group_(150–154)_, Group_(155–159)_, Group_(160–164)_, Group_(165–169)_ and Group_(170–174)_ respectively included parturients with the 145–149 cm, 150–154 cm, 155–159 cm, 160–164 cm, 165–169 cm and 170–174 cm height. There was only 1 parturient in Group_(145–149)_, who was not included for data analysis [1].

### Changes in anesthesia characteristic with height in Test group

The weight of parturients increased as the height increased (Table 1, *P* < 0.05). Other parturients’ baseline data, demographic data and concomitant disease had no statistical difference among groups with different heights (Table 1 and Table 2, *P* > 0.05). When the bupivacaine dose was altered basing on height, the occurrence rate of complications (hypotension, number of hypotensive recordings > 2, bradycardia, dyspnea, nausea, vomiting and dizziness) was not altered by the height (Table 3, *P* > 0.05). The time for sensory block to reach T8 (Time _sensory block to T8_), sensory level at 10 min after anesthesia (Sensory level_10 min_) > T4, time to complete motor block (Time _complete motor block_) and numbers of parturients with complete motor block at 10 min after anesthesia (Number _complete motor block_) did not present significant difference in parturients with different heights (Table 4, *P* > 0.05). Likewise, there were no statistical difference in quality of analgesia, quality of muscle relaxation, degree of intraoperative comfort and neonatal outcome among parturients with different heights (Table 5 and Table 6, *P* > 0.05).

**Table 1:** Parturient characteristics

	Group _(150–154)_	Group _(155–159)_	Group _(160–164)_	Group _(165–169)_	Group _(170–174)_	*χ*^*2*^ or* F*	*P*
n	22	45	42	15	2		
Age (years)	31.955 (4.359)	32.022 (5.750)	33.214 (5.846)	30.333 (4.451)	34.000 (2.828)	3.944	0.414
Weight (kg)	62.818 (13.818)	66.693 (8.114)	66.857 (6.846)	71.733 (7.583)	89.250 (11.667)^a^	5.313	0.001
BMI (kg/m^2^)	27.219 (5.912)	27.129 (3.421)	25.550 (2.595)	25.967 (2.464)	30.965 (3.920)^a^	2.066	0.089
Weeks of gestation (week)	37.455 (2.154)	37.200 (2.702)	37.857 (2.067)	37.533 (2.973)	38.000 (0.000)	3.895	0.420
Previous cesarean	13	25	20	5	2	4.923	0.295
Initial SBP (mmHg)	122.136(14.093)	126.378 (21.073)	120.548 (11.162)	118.733 (12.021)	153.500 (20.506)	4.986	0.289
Initial HR (beats/min)	89.409 (14.101)	85.689 (12.169)	89.071 (15.673)	79.067 (9.794)	99.000 (1.414)	9.406	0.052
Time from anesthesia initiation to skin incision (min)	19.546 (6.954)	19.511 (8.201)	18.214 (7.495)	19.667 (5.924)	18.000 (7.071)	2.768	0.597
Time from skin incision to delivery (min)	7.773 (4.231)	8.889 (4.657)	8.071 (5.325)	8.133 (4.868)	12.500 (3.536)	4.219	0.377
Operation duration (min)	51.364 (14.526)	56.422 (17.150)	53.595 (12.967)	63.600 (19.533)	64.500 (17.678)	6.160	0.187

^a^indicated that ANOVA was used. For other continuous data, Kruskal-Wallis H-test was used

**Table 2:** Concomitant disease of parturient

	Group _(150–154)_	Group _(155–159)_	Group _(160–164)_	Group _(165–169)_	Group _(170–174)_	*χ*^*2*^	*P*
n	22	45	42	15	2		
Hypertension	1	6	2	0	1	8.856	0.065
Diabetes	5	9	9	1	1	2.963	0.564
HGB < 90 g/L	0	3	0	0	0	5.532	0.237
Hyperthyroidism	1	0	1	0	0	2.402	0.662
Hypothyroidism	0	1	0	0	0	1.814	0.770
Abnormal liver function	0	1	0	0	0	1.814	0.770
Macrosomia	0	0	0	0	0	&	&

^&^ indicated that the *χ*^*2*^ value could not be obtained, because the value of each group was 0

**Table 3:** Incidence of side effects in parturients

	Group _(150–154)_	Group _(155–159)_	Group _(160–164)_	Group _(165–169)_	Group _(170–174)_	*χ*^*2*^	*P*
n	22	45	42	15	2		
Hypotension	2	6	6	3	0	1.245	0.871
Number of hypotensive recordings > 2	0	1	0	1	0	3.658	0.454
Dizziness	0	2	3	1	0	1.855	0.762
Nausea	0	1	2	0	0	1.980	0.739
Vomiting	0	0	1	0	0	2.016	0.733
Bradycardia	0	1	2	1	0	1.859	0.762
Dyspnea	0	0	0	0	0	&	&

^&^ indicated that the *χ*^*2*^ value could not be obtained, because the value of each group was 0

**Table 4:** Characteristics of spinal anesthesia

	Group _(150–154)_	Group _(155–159)_	Group _(160–164)_	Group _(165–169)_	Group _(170–174)_	*χ*^*2*^	*P*
n	22	45	42	15	2		
Time _sensory block to T8_ (min)	5.136 (1.781)	4.956 (1.445)	4.690 (1.423)	4.667 (1.759)	4.000 (0.000)	2.657	0.617
Sensory level_10 min_ > T4	4	6	9	5	0	3.574	0.467
Time _complete motor block_ (min)	10.833(5.576)	14.756(7.832)	11.333(6.019)	14.308(7.772)	/	4.760	0.190
Number _complete motor block_	13	17	24	7	0	7.154	0.209

/ indicated that the mean could not be obtained, because one parturient could not reach complete motor block and the sample size was 1

**Table 5:** Quality of anesthesia

	Group _(150–154)_	Group _(155–159)_	Group _(160–164)_	Group _(165–169)_	Group _(170–174)_	*χ*^*2*^	*P*
n	22	45	42	15	2		
Quality of analgesia	17	40	35	12	1	3.303	0.509
Quality of muscle relaxation	21	42	36	12	2	3.905	0.419
Degree of intraoperative comfort	19	38	33	11	2	1.976	0.740

The number of “excellent” parturients was calculated and presented in this table

**Table 6:** Neonatal outcome

	Group _(150–154)_	Group _(155–159)_	Group _(160–164)_	Group _(165–169)_	Group _(170–174)_	*χ*^*2*^	*P*
n	22	45	42	15	2		
Male	11	26	24	10	1	1.063	0.900
Weight (kg)	3.186 (0.644)	3.038 (0.574)	3.203 (0.409)	3.092 (0.723)	2.945 (0.290)	1.996	0.737
1 min Apgar score	9.909 (0.426)	9.844 (0.767)	9.929 (0.261)	9.800 (0.561)	10.000 (0.000)	1.248	0.870
5 min Apgar score	10.000 (0.000)	9.956 (0.298)	10.000 (0.000)	9.933 (0.258)	10.000 (0.000)	3.584	0.465
Blood gas analysis							
PH	7.273 (0.048)	7.276 (0.049)	7.274 (0.040)	7.290 (0.021)	7.290 (0.026)	2.121	0.713
PO_2_	18.500 (2.824)	16.844 (3.561)	17.262 (4.889)	18.933 (4.935)	20.000 (8.485)	4.505	0.342
PCO_2_	52.868 (4.494)	51.831 (6.247)	53.300 (7.006)	52.047 (5.985)	48.700 (5.657)	2.283	0.684
BE	-2.409 (2.576)	-2.422 (2.321)	-2.262 (2.220)	-1.200 (2.145)	-3.500 (0.707)	5.878	0.208

### Potential influencing factors for anesthesia characteristic

In Test group, the weight of parturients changed among five subgroups (Table 1, *P* < 0.05).The weight, height and BMI are potential influencing factors of the bupivacaine dose [19–21]. In this study, maternal hypotension was the primary outcome [1]. For analyzing potential influencing factors of anesthesia characteristic, we applied univariate and multivariate binary logistic regressions to analyze the correlation between maternal hypotension and height, weight or BMI. When the bupivacaine dose is based on height (Test group), the height, weight or BMI was not the risk factor (Table 7, univariate analysis). When the bupivacaine dose is unaltered (Conventional group), the height and weight were the potential risk factors (Table 8, univariate analysis, *P* < 0.10), but the multivariate analysis showed that only height was the independent risk factor (Table 8, *P* < 0.05).

**Table 7:** Univariate binary logistic regression analysis for potential risk factors of maternal hypotension (Test group)

Variables	OR	95% CI	*P*
Height	1.030	0.926–1.147	0.585
Weight	1.000	0.948–1.055	1.000
BMI	0.988	0.864–1.130	0.863

**Table 8:** Univariate and multivariate binary logistic regression analyses for potential risk factors of maternal hypotension (Conventional group)

Variables	Univariate			Multivariate		
	OR	95% CI	*P*	OR	95% CI	*P*
Height	0.887	0.815–0.966	0.006	0.896	0.812–0.989	0.029
weight	0.965	0.927–1.005	0.082	0.991	0.947–1.038	0.709
BMI	0.961	0.861–1.073	0.476			

## Discussion

### General data having no interference in the comparison of anesthesia characteristic among parturients with different heights

For parturients with different heights, the general data were not of statistic difference except for the weight (Table 1). Theoretically, the weight is increased with the height for single individuals [22, 23], which supports our result (Table 1). Because the BMI was similar among groups with different heights (Table 1), the statistic difference in weight is very likely to be attributed to different height. Although a study show that the bupivacaine dose is unnecessary to be changed with weight [2], the bupivacaine dose depends on weight in another study [19]. In addition, there is a study confirms that the weight only influences the bupivacaine dose in parturients with high BMI [24]. In this study, most parturients had a low BMI (Table 1). Moreover, in Test and Conventional groups, the weight was not relation with maternal hypotension (Table 7 and Table 8). Therefore, we considered weight and other general data were not influence factors for the comparison of anesthesia characteristic among five subgroups in this study.

### Algorithm of bupivacaine dose based on height being reasonable

In our last article, we preliminarily verify that the algorithm of bupivacaine dose based on height can reduce the occurrence rate of complications, and the characteristics of sensory and motor blocks is unaltered with height [1]. However, the general data does not be compared among parturients with different heights [1]. It is not thoroughly unclear whether other variables can influence the correlation between anesthesia characteristic and height, whether the algorithm of bupivacaine dose based on height is suitable.

The height has statistical correlation with vertebral column length [7]. Therefore, the height should influence the cerebrospinal fluid volume and dose of bupivacaine. However, a study shows that the height has no correlation with the bupivacaine dose [2], which is unaltered for caesarean section under spinal anesthesia [3–6]. In our study, when the bupivacaine dose is unaltered, the incidence of complication increases and characteristics of sensory or motor block is strengthened as the height decreases [1], and the height was relation with maternal hypotension (Table 8). When the bupivacaine dose increased as the height increased, the incidence of complications, characteristics of sensory or motor block and quality of anesthesia did not change with height (Table 3, Table 4 and Table 5), the correlation between maternal hypotension and height disappeared (Table 7). In this article, the general data did not influence the comparison of anesthesia characteristic among five subgroups (Table 1). That is, the height has an influence on the bupivacaine dose in caesarean section under spinal anesthesia, which is supported by more and more evidences [9, 19, 24]. The algorithm of bupivacaine dose based on height is reasonable.

Maternal hypotension can decrease the blood supply of placenta, and cause fetus hyoxemia and acidosis [4]. Our results showed that there was no statistical difference in maternal hypotension among parturients with different heights (Table. 3). In addition, even if the incidence of maternal hypotension is different, if the blood pressure returns to normal in a short time, the neonatal outcome is not influenced [1, 25]. Obesity may increase the operative time [26]. The BMI, and time from anesthesia initiation to delivery and time from uterine incision to delivery are associated with umbilical arterial pH [27, 28]. However, in this study, the BMI, time from anesthesia initiation to skin incision and time from skin incision to delivery were not statistically different among parturients with different heights (Table 1). Based on the above analysis, it is reasonable that the neonatal outcome did not change with height in this article.

### Strengths and limitations

By the heights, we grouped the parturients, who was executed the algorithm of bupivacaine dose based on height. We carried out the comparisons among subgroups for all variables, and univariate and multivariate binary logistic regressions to reanalyze the interference factor for the bupivacaine dose and to investigate the rationality of this algorithm of bupivacaine dose based on height.

This article has four limitations. Firstly, in Group_(170–174)_, the number of parturients was too small, which may influence the results. Secondly, for women in America or Europe, the body shape and height are different from both in China. It is unclear whether this algorithm of bupivacaine dose based on height is suitable. Thirdly, isobaric bupivacaine was used in this study. Hypobaric or hyperbaric bupivacaine may be not suitable to be adjusted by using this dosing algorithm based on height [29–31]. Fourthly, in this study, although we included obese parturients at recruitment, we recruited very few parturients with high BMI (Table 1). This dosing algorithm based on height may be not suitable for the obese parturients.

## Conclusions

The height is the interference factor for the bupivacaine dose, but the weight and BMI are not. The dosing algorithm based on height is reasonable for adjusting the bupivacaine dose in this article.

## Data Availability

The datasets analyzed during the current study are available from the corresponding author upon reasonable request.

## Abbreviations

BMI: Body mass index
CSE: Combined spinal-epidural anesthesia
Group_(145–149)_, Group_(150–154)_, Group_(155–159)_, Group_(160–164)_, Group_(165–169)_ or Group_(170–174)_: Group included parturients with the 145–149 cm, 150–154 cm, 155–159 cm, 160–164 cm, 165–169 cm or 170–174 cm height in Test group
HR: Heart rate
Number _complete motor block_: Numbers of parturients with complete motor block at 10 min after anesthesia
SBP: Systolic blood pressure
Sensory level _10 min_: Sensory level at 10 min after anesthesia
T4, T5, T6 or T8: Dermatome level dominated by the fourth, five, six or eight thoracic nerve
Time _complete motor block_: Time to complete motor block
Time _sensory block to T8_: Time for sensory block to reach T8
